# The Association Between Negative Emotion and Suicidal Ideation in Chinese Migrant Workers: A Chain Mediation Model of Meaning in Life and Social Connectedness

**DOI:** 10.3390/bs16071120

**Published:** 2026-07-04

**Authors:** Chen Hong, Lili Liu, Ting Pan, Weiman Yan, Yu Wang, Ning Chen, Wei Liu

**Affiliations:** 1 School of Psychology, Shanghai Normal University, Shanghai 200234, China; 1000486373@smail.shnu.edu.cn (C.H.); 1000567144@smail.shnu.edu.cn (W.Y.); 1000567142@smail.shnu.edu.cn (Y.W.); 2 Mental Health Education Center, Yiyang Medical College, Yiyang 413000, China; liulili00000@163.com; 3 Mental Health Education and Counseling Center, Yiyang Vocational & Technical College, Yiyang 413000, China; panting20111@163.com

**Keywords:** suicidal ideation, migrant workers, negative emotion, social connectedness, meaning in life, interpersonal theory of suicide

## Abstract

Negative emotion has been identified as a critical risk factor for suicidal ideation, yet the interpersonal psychological mechanisms underlying this association remain poorly understood among Chinese migrant workers, a population characterized by social marginalization and heightened vulnerability. Guided by the interpersonal theory of suicide, the present study examined the relationship between negative emotion and suicidal ideation and further investigated whether meaning in life and social connectedness functioned as sequential mediators in this relationship. A total of 739 migrant workers from Beijing and Shanxi Province completed self-report questionnaires assessing negative emotion, meaning in life, social connectedness, and suicidal ideation. The results indicated that negative emotion was positively associated with suicidal ideation, whereas higher levels of meaning in life and social connectedness were associated with lower suicidal ideation. Mediation analyses demonstrated that meaning in life and social connectedness partially mediated the link between negative emotion and suicidal ideation, both individually and sequentially. These findings elucidate an interpersonal psychological pathway through which negative emotion may be associated with heightened suicidal ideation by eroding intrapersonal meaning and interpersonal connectedness. Interventions aimed at enhancing meaning in life and strengthening social connectedness may hold promise for mitigating suicide risk among this vulnerable population.

## 1. Introduction

Migrant workers in China are individuals who work and reside in urban areas without obtaining an urban household registration (hukou) (J. Chen, 2011). This population emerged as a result of China’s rapid urbanization process, which, since the 1980s, has been accompanied by large-scale migration of rural laborers into cities (Zhong et al., 2016). According to the 2023 Migrant Workers Monitoring Survey Report released by the National Bureau of Statistics of China (2024), the total number of migrant workers reached 297.35 million in 2023. Due to institutional constraints and structural inequalities, migrant workers remain disadvantaged compared to other urban residents in areas such as employment, social security, children’s education, and housing (Wong et al., 2007), placing them at elevated risk for mental health problems. This institutionalized urban-rural social isolation, combined with precarious employment, lack of social security, and prolonged family separation, creates a unique sociocultural environment that places them at an elevated risk for severe psychological distress and suicidal ideation (J. Li & Rose, 2017). Therefore, examining suicidal ideation within this specific localized context is both theoretically necessary and practically urgent.

Suicidal ideation warrants particular attention among the mental health challenges faced by migrant workers (Zhong et al., 2019). Suicidal ideation refers to thoughts about or plans for suicide (Harmer et al., 2024) and is a critical and distinct risk factor for suicidal behavior (Brown et al., 2000). Evidence indicates that the lifetime prevalence of suicidal ideation among Chinese migrant workers is as high as 12.8% (L. Chen et al., 2020), significantly exceeding the 3.9% reported in the general population of mainland China (Cao et al., 2015). These findings highlight the heightened vulnerability of migrant workers to suicidal ideation (Zhong et al., 2019). Accordingly, examining the mechanisms underlying suicidal ideation among migrant workers and identifying effective intervention pathways holds important theoretical and practical value.

Negative emotion has been consistently identified as a well-established risk factor for suicidal ideation (Liu & Miller, 2014). Intense negative emotional experiences are closely linked to suicidal ideation, as they may exacerbate psychological distress and maladaptive cognitive processes (He et al., 2023). According to the interpersonal theory of suicide, suicidal ideation is closely associated with perceived burdensomeness, reflected in doubts about one’s purpose and value in life, as well as thwarted belongingness, which underscores the critical role of social connectedness in the development of suicidal thoughts (Van Orden et al., 2010; S. Li et al., 2024). Building on these findings, the present study focuses on the roles of meaning in life and social connectedness in the association between negative emotion and suicidal ideation.

### 1.1. The Relationship Between Negative Emotion and Suicidal Ideation

Negative emotion refers to a broad affective condition encompassing multiple emotional experiences, including depression, anxiety, tension, and subjective distress. This affective state is often elicited by adverse life events or interpersonal frustration and is closely associated with a range of mental health outcomes (Lovibond & Lovibond, 1995; Liu & Miller, 2014).

A substantial body of research has identified negative emotion as one of the most widely recognized risk factors for suicidal ideation (X. Zhang et al., 2017). Notably, suicidal ideation may, in turn, intensify negative emotional experiences, thereby creating a self-reinforcing cycle in which emotional distress contributes to suicidal thoughts, which then further exacerbate psychological suffering (Al-Dajani & Uliaszek, 2021). Y. Wang et al. (2025), using network analysis, constructed networks of depressive and anxiety symptoms among migrant workers in Shenzhen and found that “uncontrollable worry” was most directly linked to suicidal ideation, suggesting a close association between a broad range of negative emotions and suicidal thoughts.

The interpersonal theory of suicide (Van Orden et al., 2010) provides a theoretical framework for understanding this relationship. According to the theory, negative emotional states are often accompanied by thwarted belongingness and interpersonal distress, which, when persistent, increase the likelihood of suicidal ideation (Chu et al., 2017). Taken together, negative emotion and suicidal ideation are tightly and complexly connected: negative emotion is not only a major risk factor for suicidal ideation but also a core driving force in its formation and maintenance. Based on this reasoning, the present study proposes.

**Figure 1:**
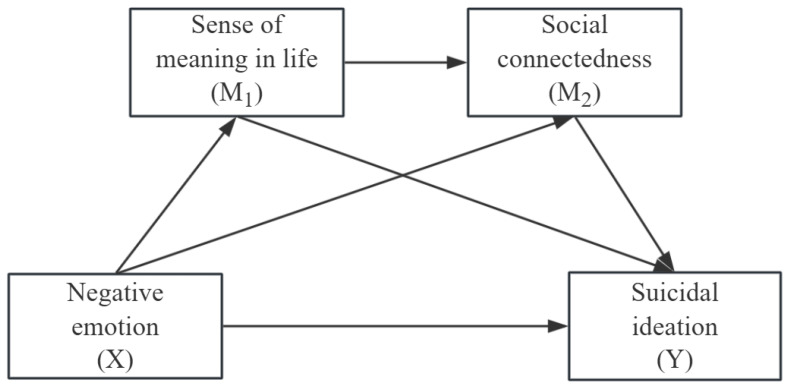
Hypothesized chain mediation model.

**Hypothesis** **1.** *Negative emotion significantly predicts suicidal ideation (Figure 1).*

### 1.2. Meaning in Life as a Potential Mediator 

Meaning in life refers to individuals’ subjective perception that their lives possess value, purpose, and significance to others (King & Hicks, 2021). Prior research demonstrates that lack of meaning in life is positively associated with negative emotional states such as depression and anxiety (Steger et al., 2006). Negative emotions tend to reinforce maladaptive cognitions and hopelessness, undermining individuals’ sense of purpose and self-worth, ultimately diminishing meaning in life (Park, 2010). This pattern has also been observed among migrant workers, as experiences of exclusion and discrimination intensify feelings of burdensomeness and reduce meaning in life (J. Li & Rose, 2017). According to the interpersonal theory of suicide, feelings of burdensomeness and thwarted belongingness (i.e., low meaning in life) contribute to heightened suicidal ideation (Van Orden et al., 2010). Conversely, increasing meaning in life has been identified as a key protective factor against suicidal ideation (Heisel & Flett, 2014). Thus, meaning in life may serve as a mediator in the relationship between negative emotion and suicidal ideation.

**Hypothesis** **2.** *Meaning in life mediates the link between negative emotion and suicidal ideation (Figure 1).*

### 1.3. Social Connectedness as a Potential Mediator 

Social connectedness refers to individuals’ perceived sense of connection, closeness, and support from others and from social groups—such as having trusted friends, feeling loved, feeling protected, and experiencing a sense of belonging (Lee & Robbins, 1995, 1998). Extensive research has shown that social connectedness is associated with lower levels of common psychological symptoms, such as depression and anxiety (Lee & Robbins, 1998), and more broadly protects against a range of psychological and behavioral problems by promoting higher levels of well-being and adaptive functioning (Cacioppo & Cacioppo, 2014; Holt-Lunstad, 2021, 2024). Positive emotions enhance social connectedness by broadening individuals’ cognitive and behavioral capacities and fostering prosocial behavior and gratitude (Algoe, 2012; Fredrickson, 2001), whereas negative emotions, such as anxiety and depression, tend to reduce social interaction and weaken individuals’ sense of belonging (Long et al., 2022). Individuals with low social connectedness are at higher risk of suicidal ideation and behavior (Bourassa et al., 2024). Within the interpersonal theory of suicide, feelings of social disconnection are core manifestations of thwarted belongingness and are closely tied to suicidal thoughts (Van Orden et al., 2010). Thus, social connectedness may mediate the relationship between negative emotion and suicidal ideation.

**Hypothesis** **3.** *Social connectedness significantly mediates the relationship between negative emotion and suicidal ideation (Figure 1).*

### 1.4. The Mediating Role of Meaning in Life and Social Connectedness

Meaning in life and social connectedness are also closely interconnected. Individuals with a strong sense of meaning in life tend to be more engaged and attractive in social interactions, making it easier for them to establish and maintain positive interpersonal relationships (Stillman et al., 2011). Meaning in life promotes individuals’ motivation to seek social connections (Stavrova & Luhmann, 2016). The interpersonal theory of suicide posits that thwarted belongingness (i.e., low social connectedness) and perceived burdensomeness are two central interpersonal risk factors that jointly contribute to the emergence of suicidal desire (Van Orden et al., 2010). As a psychological protective factor, meaning in life helps foster positive emotions and alleviate loneliness, anxiety, and depression (B. Zhang et al., 2024). Crucially, while the interpersonal theory of suicide often treats perceived burdensomeness and thwarted belongingness as parallel risk factors (Van Orden et al., 2010), meaning in life and social connectedness may operate sequentially among migrant workers. Grounded in the “meaning as magnetic force” perspective (Stillman et al., 2011), meaning in life functions as a critical intrapersonal resource that motivates social engagement. When negative emotions deeply erode this intrapersonal sense of meaning, it often manifests as depressive social withdrawal. This existential depletion subsequently damages their motivation and capacity to maintain interpersonal relationships, ultimately resulting in reduced social connectedness. Based on this.

**Hypothesis** **4.** *Meaning in life and social connectedness play a significant chain mediating role in the relationship between negative emotion and suicidal ideation (Figure 1).*

## 2. Methods

### 2.1. Participants

A convenience sampling strategy was adopted to recruit migrant workers from construction, manufacturing, and service enterprises located in Beijing and Shanxi Province. In accordance with the definition of migrant workers outlined in Article 2 of the Regulations on Ensuring the Payment of Wages to Migrant Workers (State Council of the People’s Republic of China, Decree No. 724), participants were required to meet two core criteria: (1) possessing rural household registration (hukou), and (2) providing labor for an employing unit. In addition, we included the requirement that participants must be providing labor in an urban or township area, regardless of full-time or part-time status.

This sampling method and geographic restriction present inherent limitations in representativeness. Data collection took place between May and June 2022. Initially, 800 electronic questionnaires were distributed. Participants completed the questionnaire on their own mobile devices, and trained research assistants provided standardized instructions and procedural guidance when necessary. All participants were informed of the purpose and procedures of the study and voluntarily signed an informed consent form prior to participation. Participants were assured that their responses would remain confidential. Questionnaires were excluded if they exhibited patterned or non-differentiated responding, contained substantial missing data, or were completed in an unreasonably short time, suggesting insufficient engagement with the survey. Ethical approval was obtained from the relevant institutional ethics committee prior to data collection. After excluding questionnaires that exhibited patterned responding or were completed in an unreasonably short time (e.g., under 180 s), 739 valid responses were retained, yielding an effective response rate of 92.3%. It is important to note that due to the constraints of the initial survey design, comprehensive demographic data—such as marital status, educational attainment, specific monthly income, and length of migrant work experience—were unfortunately not collected.

A total of 739 eligible migrant workers completed the questionnaire, including 683 males (92.42%) and 56 females (7.58%). Participants ranged in age from 18 to 46 years, with a mean age of 24.06 years (SD = 4.27). Demographic characteristics of the sample are presented in Table 1.

### 2.2. Measures

#### 2.2.1. Negative Emotion

Negative emotion was measured using the Negative Affect subscale of the Chinese version of the Positive and Negative Affect Schedule (PANAS), originally developed by Watson et al. (1988) and revised by W. Zhang et al. (2004). The subscale consists of 10 adjectives describing negative emotional states (e.g., “upset,” “guilty”), rated on a five-point Likert scale (1 = “very slightly or not at all,” 5 = “extremely”). Higher scores indicate stronger negative emotional intensity. In this study, the scale demonstrated good internal consistency (Cronbach’s α = 0.916).

#### 2.2.2. Meaning in Life

Meaning in life was assessed using the Chinese version of the Meaning in Life Questionnaire (C-MLQ), developed by Steger et al. (2006) and adapted by M. C. Wang and Dai (2008). The scale contains 10 items measuring two dimensions: Presence of Meaning (e.g., “My life has a clear sense of purpose”) and Search for Meaning (e.g., “I am seeking a purpose or mission for my life”). Items are rated on a seven-point Likert scale (1 = “absolutely untrue,” 7 = “absolutely true”). Higher scores represent a stronger sense of meaning in life. The internal consistency coefficient in the present study was 0.865.

#### 2.2.3. Social Connectedness

Social connectedness was measured using the Social Connectedness Scale based on Wildschut et al. (2006). The scale includes four items capturing trust (“I can trust other people”), perceived love (“I am loved”), perceived protection (“I am protected”), and emotional connectedness (“I feel connected with the people I love”). Items are rated on a seven-point Likert scale (1 = “strongly disagree,” 7 = “strongly agree”). Higher scores indicate stronger perceived social connectedness. The internal consistency coefficient in the present study was 0.900.

#### 2.2.4. Suicidal Ideation

Suicidal ideation was measured using the Suicide Ideation Self-Rating Scale (SIOSS) developed by Xia et al. (2007). The scale consists of 26 items, each rated dichotomously as “yes” (1) or “no” (0) (e.g., “I have had thoughts of ending my life”). Higher scores indicate stronger suicidal ideation. In the present study, the scale showed acceptable reliability (Cronbach’s α = 0.786).

### 2.3. Data Analysis

Data analysis was conducted using SPSS version 27.0 (IBM Corp., Armonk, NY, USA) and the PROCESS macro (version 4.1). Given that all variables were assessed through self-report questionnaires, Harman’s single-factor test was used to examine potential common method bias. Exploratory factor analysis of all measurement items indicated that the first unrotated factor accounted for 24.975% of the total variance, which was below the commonly accepted threshold of 40%, indicating that common method bias was unlikely to be a serious problem in this study.

Descriptive statistics and correlation analyses were first performed. Subsequently, Model 6 of the PROCESS macro was employed to test the proposed chain mediation model, with negative emotion as the independent variable, suicidal ideation as the dependent variable, and meaning in life and social connectedness as mediators. A bootstrap sampling method with 5000 resamples was used to estimate indirect effects and generate 95% confidence intervals. An indirect effect was considered statistically significant if its confidence interval did not include zero.

## 3. Results

### 3.1. Descriptive Statistics and Correlation Analysis

Table 2 presents the descriptive statistics and correlations among all variables. Negative emotion was significantly negatively correlated with meaning in life (*r* = −0.128, *p* < 0.01) and social connectedness (*r* = −0.337, *p* < 0.01), and positively correlated with suicidal ideation (*r* = 0.414, *p* < 0.01). In addition, meaning in life was significantly positively correlated with social connectedness (*r* = 0.513, *p* < 0.01) and significantly negatively correlated with suicidal ideation (*r* = −0.320, *p* < 0.01). Social connectedness was also significantly negatively correlated with suicidal ideation (*r* = −0.528, *p* < 0.01).

These results preliminarily indicate that negative emotion may increase the risk of suicidal ideation, while meaning in life and social connectedness may serve as protective factors.

### 3.2. Negative Emotion Predicts Suicidal Ideation

To examine Hypothesis 1, we tested whether negative emotion directly predicts suicidal ideation. After controlling for the mediating variables, the overall regression model significantly predicted suicidal ideation (*R*^2^ = 0.347, *F*(3, 735) = 130.150, *p* < 0.001), and negative emotion remained a significant predictor of suicidal ideation (*β* = 0.270, *p* < 0.001). This supports Hypothesis 1 and suggests that higher levels of negative emotion are associated with greater suicidal ideation among migrant workers.

### 3.3. Mediating Effect of Meaning in Life

To test Hypothesis 2, we examined whether meaning in life mediates the association between negative emotion and suicidal ideation. The regression model with negative emotion predicting meaning in life showed a significant fit (*R*^2^ = 0.016, *F*(1, 737) = 12.327, *p* < 0.001). Results showed that the indirect effect of meaning in life was significant (*β* = 0.011, 95% CI [0.001, 0.023]), indicating that negative emotion was associated with lower meaning in life, which in turn predicted higher suicidal ideation. Therefore, Hypothesis 2 was supported.

### 3.4. Mediating Effect of Social Connectedness

The mediating role of social connectedness was examined to test Hypothesis 3. The regression model predicting social connectedness also demonstrated a significant fit (*R*^2^ = 0.338, *F*(2, 736) = 188.118, *p* < 0.001). Results demonstrated a significant indirect effect through social connectedness (*β* = 0.109, 95% CI [0.077, 0.143]). This suggests that negative emotion was associated with reduced social connectedness, which in turn increased suicidal ideation. Hypothesis 3 was therefore supported.

### 3.5. Chain Mediating Effect of Meaning in Life and Social Connectedness

Finally, Hypothesis 4 proposed that meaning in life and social connectedness would sequentially mediate the relationship between negative emotion and suicidal ideation. The chain mediation pathway was significant (*β* = 0.024, 95% CI [0.008, 0.042]). These findings indicate that negative emotion lowers meaning in life, which subsequently diminishes social connectedness, ultimately leading to higher suicidal ideation. Thus, Hypothesis 4 was supported.

**Figure 2:**
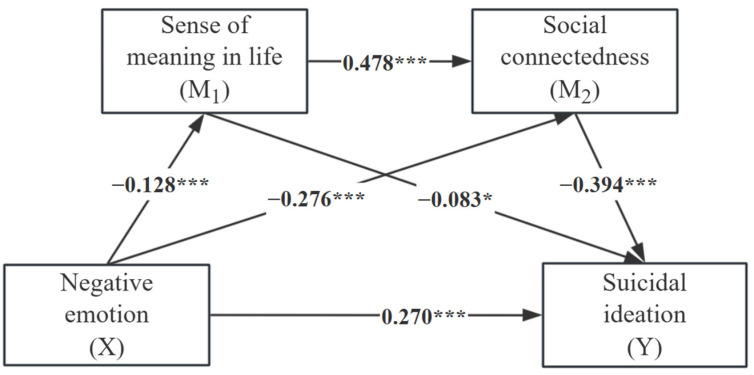
A chain mediation model for negative emotion, sense of meaning in life, social connectedness, and suicidal ideation. Note. * *p* < 0.05; *** *p* < 0.001.

Table 3 presents the specific mediation effects, and Figure 2 illustrates the chain mediation model.

## 4. Discussion

The present study examined the relationships among negative emotion, meaning in life, social connectedness, and suicidal ideation among Chinese migrant workers, and further tested the mediating roles of meaning in life and social connectedness. Consistent with our hypotheses, negative emotion was positively associated with suicidal ideation, while meaning in life and social connectedness were negatively associated with suicidal ideation. Moreover, meaning in life and social connectedness each independently mediated the relationship between negative emotion and suicidal ideation, and a significant serial mediation effect through meaning in life and social connectedness was also observed.

### 4.1. Negative Emotion and Suicidal Ideation

The present study found that negative emotion significantly predicted suicidal ideation, which is consistent with previous research (Qiu et al., 2017). Migrant workers in China are often exposed to deep-rooted structural exclusion within the social system (J. Li & Rose, 2017). Compared with other social groups, they are more likely to encounter discrimination and unfair treatment and frequently experience social isolation (Ren et al., 2018). These chronic and cumulative stressors substantially heighten their risk of developing negative emotions (T. Yang et al., 2012). According to the interpersonal theory of suicide, negative emotion and adverse interpersonal circumstances may give rise to thwarted belongingness; when such rejection or isolation persists over time, suicidal ideation may emerge (Van Orden et al., 2010; Chu et al., 2017). Consequently, the prevalence of suicidal ideation is relatively high among migrant workers (Lau et al., 2012), accompanied by multiple mental health problems (T. Yang et al., 2012).

### 4.2. The Mediating Role of Meaning in Life

The present study found that meaning in life significantly mediated the relationship between negative emotion and suicidal ideation. This mediating effect is consistent with prior research showing that meaning in life is negatively associated with aversive psychological experiences (e.g., intense negative emotion) (Arslan & Yıldırım, 2021; Costin & Vignoles, 2020) and that individuals with a stronger sense of meaning in life tend to report lower levels of suicidal ideation (Yıldırım et al., 2022). Migrant workers frequently live far away from their families and friends (Xiao et al., 2022), and many work in low-skilled and low-wage positions (Wong et al., 2007). These circumstances often engender feelings of inferiority, helplessness, and worthlessness. In addition, a substantial proportion of migrant workers engage in unstable employment, including informal, temporary, or daily-wage jobs, which restrict their opportunities for leisure and their ability to fulfill family responsibilities (Cai et al., 2021; Woo, 2022). Prior research shows that unstable employment combined with high social pressure can heighten individuals’ perceived workload and social burden, thereby contributing to emotional exhaustion and diminished self-worth (Xu et al., 2023). Under such persistent strain and self-deprecating experiences, migrant workers may gradually internalize these external pressures as interpersonal self-negation, manifested as the perception of being a burden to others. Perceived burdensomeness is a core interpersonal risk factor that has been shown to directly contribute to suicidal desire (Van Orden et al., 2010). In this context, the significant mediating role of meaning in life identified in the present study highlights how negative emotional experiences, rooted in perceived burdensomeness, translate into increased suicidal ideation by diminishing individuals’ perceived value and purpose in life. Accordingly, these findings underscore the importance of addressing the psychological needs of migrant workers within their work environments (Mo et al., 2022). It is also worth noting the relatively weak bivariate correlation between negative emotion and meaning in life (*r* = −0.128) found in this study. This suggests that while negative emotional states do erode one’s existential purpose, meaning in life among Chinese migrant workers may be concurrently sustained by strong cultural or familial duties, such as the drive to provide financial support for left-behind children (Xiao et al., 2022). Nevertheless, within the broader multivariate framework, this pathway remains a statistically and theoretically relevant component of the cascade toward suicidal ideation.

### 4.3. The Mediating Role of Social Connectedness

The findings also showed that social connectedness significantly mediated the relationship between negative emotion and suicidal ideation. This aligns with previous research suggesting that negative emotional states—such as anxiety and depression—may reduce social interaction and subsequently undermine social connectedness (Long et al., 2022). Individuals with higher perceived social connection demonstrate a significantly lower risk of suicidal ideation (Celik & Cakar, 2025). Migrant workers frequently face stigma, discrimination, and identity conflict arising from urban social exclusion, which directly damages core components of social connectedness—namely, belongingness and perceived support (J. Li & Rose, 2017; Y. Yang et al., 2023). This underscores the urgency of enhancing social connectedness among this population. According to the interpersonal theory of suicide, unmet belongingness represents another central interpersonal risk factor associated with suicidal desire (Van Orden et al., 2010). The mediating role of social connectedness observed in this study suggests that perceived exclusion and isolation may directly weaken migrant workers’ social connectedness, thereby increasing the likelihood of suicidal ideation. Additionally, migrant workers are often situated on the margins of urban social networks, frequently experiencing a lack of intimate relationships, inadequate family support, and fragile social support systems, all of which contribute to diminished self-esteem (B. Wang et al., 2010; Wong & Leung, 2008). These factors further constrain opportunities for developing meaningful social connections. The present findings therefore highlight the importance of enhancing social connectedness among migrant workers through systematic interventions as a means of reducing suicide risk.

### 4.4. The Chain Mediating Role of Meaning in Life and Social Connectedness

Finally, the study found that meaning in life and social connectedness jointly exerted a significant chain-mediating effect between negative emotion and suicidal ideation. Meaning in life not only reflects an internal experience of purpose and value but also is associated with greater engagement in social interactions and interpersonal processes that facilitate the development and maintenance of social relationships (Stillman et al., 2011; King & Hicks, 2021). Stillman et al. (2011) demonstrated that individuals with greater meaning in life exhibit higher interpersonal appeal and receive more positive social responses, thereby developing stronger social connectedness. Longitudinal evidence from Stavrova and Luhmann (2016) also indicates that meaning in life significantly predicts subsequent levels of social connectedness, functioning as an important antecedent of social engagement and integration. Therefore, when negative emotion undermines meaning in life, individuals may be more likely to withdraw socially, leading to reduced social connectedness; weakened social connectedness, in turn, further increases the risk of suicidal ideation (Reynolds et al., 2022). Consistent with existing theoretical perspectives, the present findings indicate that meaning in life and social connectedness together serve as key protective factors against suicidal ideation among migrant workers (Kleiman et al., 2013; Celik & Cakar, 2025). Crucially, these findings offer a nuanced, localized expansion of the Interpersonal Theory of Suicide (IPTS). While the original IPTS framework posits perceived burdensomeness and thwarted belongingness as distinct, parallel constructs (Van Orden et al., 2010), our serial mediation model suggests a sequential vulnerability among migrant workers. For this marginalized population, the structural erosion of self-worth and life meaning (an internal existential crisis analogous to perceived burdensomeness) acts as the catalyst that subsequently damages their motivation or capacity to engage in interpersonal relationships, thereby precipitating thwarted belongingness. Taken together, these findings underscore the essential role of enhancing meaning in life and strengthening social connectedness in interventions aimed at reducing suicide risk and improving the psychological well-being of this vulnerable population.

### 4.5. Implications

The findings of this study offer clear directions for interventions aimed at reducing suicidal ideation among migrant workers. First, emotion regulation programs tailored to address common occupational stressors among this population, such as long working hours, job insecurity, and high work intensity, could help mitigate the harmful effects of negative emotion. Secondly, when evaluating the mediation pathways, the indirect effect through social connectedness (*β* = 0.109) was noticeably stronger than through meaning in life (*β* = 0.011). This suggests that for migrant workers, immediate interpersonal relational factors act as more potent buffers against suicidal ideation than intrapersonal existential reflection. Therefore, interventions should prioritize relationship building. Given the high mobility and marginalized status of this population, localized and practical interventions are essential. For instance, enterprises could establish peer-support networks or ‘workplace mentoring’ systems directly within factory dormitories or construction sites to immediately address thwarted belongingness. Furthermore, considering the hukou-related barriers to accessing formal urban psychiatric care, leveraging mobile-based digital mental health screening and tele-counseling could provide accessible, low-barrier support. In summary, integrated interventions that concurrently address emotional, meaning-based, and relational resources are likely to be most effective for this population.

### 4.6. Limitations and Future Directions

Several severe limitations must be addressed. First, the cross-sectional design strictly precludes causal inference. A reverse causality is highly plausible; the presence of suicidal ideation could actively drive social withdrawal and a collapse of life meaning. Future research must employ longitudinal or cross-lagged panel models to verify the sequential directionality. Second, the reliance on convenience sampling from only Beijing and Shanxi Province resulted in severe selection bias. The sample’s demographic characteristics are extremely skewed, with an overwhelming majority being male (92.42%) and predominantly young. Consequently, these findings lack geographical and demographic representativeness and cannot be generalized to the broader national migrant worker population. Extreme caution should be exercised before applying these conclusions to female or older migrant workers. Third, this study failed to control for key confounding demographic variables. Crucial data—including marital status, educational attainment, length of migrant work experience, and specific income—were omitted during data collection. The failure to include these covariates, alongside gender and age, in the mediation model may have led to an overestimation of the effect sizes, which limits the robustness of the results. Future research must strictly incorporate and control for these sociodemographic variables.

## 5. Conclusions

Grounded in the Interpersonal Theory of Suicide, this study reveals a chain mediating mechanism through which negative emotion is associated with suicidal ideation among Chinese migrant workers. The findings demonstrate that negative emotion not only directly predicts suicidal ideation but also indirectly increases it through two pathways: by reducing meaning in life and by weakening social connectedness. More importantly, lowered meaning in life further undermines social connectedness, forming a sequential pathway linking negative emotion to suicidal ideation. These results highlight the importance of enhancing meaning in life and strengthening social connectedness as key psychological resources for mitigating the impact of negative emotion and reducing suicidal risk in this population.

## Figures and Tables

**Table 1:** Demographic characteristics of participants (*n* = 739).

Demographic Variable	Category	Frequency	Percentage (%)
Age	18–20	149	20.16
	21–30	520	70.36
	31–40	68	9.20
	41–46	2	0.28
Gender	Male	683	92.42
	Female	56	7.58
Total		739	100

**Table 2:** Descriptive statistics and zero-order correlations.

Variables	M (SD)	1	2	3	4
**1 Negative emotion**	1.982 ± 0.654				
**2 Sense of meaning in life**	5.442 ± 1.047	−0.128 **			
**3 Social connectedness**	6.012 ± 1.074	−0.337 **	0.513 **		
**4 Suicidal ideation**	0.092 ± 0.137	0.414 **	−0.320 **	−0.528 **	

Note. *n* = 739. ** *p* < 0.01.

**Table 3:** Mediation effects of sense of meaning in life and social connectedness.

Mediation Pathways	*β*	Boot SE	95% CI [Lower, Upper]
Total	0.144	0.021	[0.101, 0.184]
Ind1	0.011	0.006	[0.001, 0.023]
Ind2	0.109	0.017	[0.077, 0.143]
Ind3	0.024	0.009	[0.008, 0.042]

Note. *β* = standardized regression coefficient; SE = standard error; CI = confidence interval. Ind1 = Negative emotion → Sense of meaning in life → Suicidal ideation. Ind2 = Negative emotion→ social connectedness → Suicidal ideation. Ind3 = Negative emotion → Sense of meaning in life → social connectedness → Suicidal ideation.

## Data Availability

The data presented in this study are available on request from the corresponding author. The data are not publicly available due to privacy and ethical restrictions.
